# Draft Genome Sequence of *Klebsiella pneumoniae* UCD-JA29 Isolated from a Patient with Sepsis

**DOI:** 10.1128/genomeA.00234-16

**Published:** 2016-05-05

**Authors:** Alexandra Alexiev, David A. Coil, Guillaume Jospin, Jonathan A. Eisen, Jason Y. Adams

**Affiliations:** a University of California Davis Genome Center, Davis, California, USA; b Department of Evolution and Ecology, University of California Davis, Davis, California, USA; c Department of Medical Microbiology and Immunology, University of California Davis, Davis, California, USA; d Division of Pulmonary, Critical Care, and Sleep Medicine, Department of Internal Medicine, University of California Davis School of Medicine, Sacramento, California, USA

## Abstract

Here, we present the 6,155,188-bp draft genome sequence of *Klebsiella pneumoniae* UCD-JA29, isolated from blood cultures from a patient with sepsis at the University of California, Davis Medical Center in Sacramento, California, USA.

## GENOME ANNOUNCEMENT

Sepsis is caused by a systemic inflammatory response to bacteria and bacterial toxins in the bloodstream (1). Sepsis can result in multiple organ failure, which is associated with high rates of morbidity and mortality (2). Over the past 30 years, rates of sepsis have increased among hospitalized patients and sepsis-related costs have increased substantially. In 2009, sepsis was the most expensive reason for hospitalization, amounting to $15.4 billion in hospital costs (4.3% of all hospital costs), and was the sixth most common cause of hospitalization (1). *Klebsiella pneumoniae* is a Gram-negative, nonmotile, rod-shaped opportunistic pathogen that is common in nature and is frequently found in the human nasopharynx and intestines (3). In addition, it is one of the most common causes of Gram-negative multidrug-resistant nosocomial infections (4). Little is known about how host susceptibility and virulence-associated factors relate to the severity of human sepsis. This project aimed to use a genomics-based approach to identify pathogen genomic features associated with high severity of illness in patients with sepsis. *Klebsiella pneumoniae* UCD-JA29 was isolated from a blood culture from a patient with sepsis at the University of California, Davis Medical Center in Sacramento, California, USA.

*K. pneumoniae* UCD-JA29 was isolated from an overnight-plated subculture of initial liquid blood cultures obtained in the course of routine clinical practice and stored in a biorepository at −80°C. A single colony was then grown in LB broth at 37°C and was subsequently used for genomic DNA extraction using a Mo Bio Powersoil DNA extraction kit (Mo Bio, Carlsbad, CA, USA). Illumina 300-bp paired-end libraries were produced using an Illumina TruSeq kit (Illumina, San Diego, CA, USA) and sequenced on an Illumina MiSeq.

Sequencing/assembly generated 1,237,876 reads and approximately 37.9× coverage. The genome size was 6,155,188 bp, and the GC content was 55.7%. All sequence processing and assembly of the Illumina reads were performed using the A5-miseq assembly pipeline (5, 6). Automated annotation was performed using the RAST annotation server (7), which identified 5,806 predicted protein coding sequences and 76 noncoding RNAs.

### Nucleotide sequence accession numbers.

This genome sequence has been deposited at DDBJ/EMBL/GenBank under the accession number LQHH00000000. The version described in this paper is the first version, LQHH00000000.1.
